# Antibacterial Efficacy of *Pseudomonas aeruginosa* Bacteriophages on a *Drosophila* Infection Model

**DOI:** 10.3390/pathogens15040411

**Published:** 2026-04-10

**Authors:** Karel Petrzik, Sára Brázdová

**Affiliations:** Biology Centre Academy of Sciences of the Czech Republic, Institute of Plant Molecular Biology, Branišovská 31, 370 05 České Budějovice, Czech Republic; sara.souckova@umbr.cas.cz

**Keywords:** *Pseudomonas aeruginosa*, animal model, new virus genus, phagotherapy, chronic infection

## Abstract

*Pseudomonas aeruginosa* is a widespread pathogen that causes acute and chronic diseases in various organisms, including humans. Treating this antibiotic-resistant bacterium is challenging, so alternative or supplementary treatment strategies are desirable. Six novel bacteriophages specific to *P. aeruginosa* were isolated and classified into the genera *Septimatrevirus*, *Kochitakasuvirus*, *Bruynoghevirus*, and a new, unnamed genera related to *Napahavirus*, and *Kantovirus*. Their genomes were annotated and further characterized. We used the *Drosophila melanogaster* insect model to predict the efficacy of the phages in terms of their curative function on other organisms. Flies were chronically infected by feeding them bacteria and were subsequently treated with individual bacteriophages. The results of the Kaplan–Meier survival test revealed differences in phage efficacy and supported the hypothesis that the phages had a curative effect. These mentioned phages extended the flies’ lifespan.

## 1. Introduction

*Pseudomonas aeruginosa* is a motile, Gram-negative bacterium that is ubiquitous in soil, on moist surfaces, and in freshwater. It inhabits humans and other vertebrates, as well as insects, nematodes, and plants. It is widely accepted that soil and plants act as reservoirs for the bacterium. While some strains from the rhizosphere have been shown to exhibit beneficial traits for the plants, others cause wilt, severe soft-rot-like symptoms, basal stem rot, and leaf spots in various crops [1]. In contrast, *P. aeruginosa* is also one of the most important animal and human pathogens in the ESKAPE group (*Enterococcus faecium*, *Staphylococcus aureus*, *Klebsiella pneumoniae*, *Acinetobacter baumannii*, *Pseudomonas aeruginosa*, and *Enterobacter* sp.), as defined by the WHO [2]. The infection in animals and humans manifests in the form of dermatitis, soft tissue and bone infection, with acute and chronic presentations. Chronic manifestations are enhanced by the ability of the bacteria to form biofilms. Due to the presence of *P. aeruginosa* in different ecosystems, the genome of *P. aeruginosa* varies greatly in size from 5.5 Mbp to 7 Mbp, due to the presence of prophages, transposons, and insertion sequences [3]. These horizontally transmissible elements contribute to the virulence and antibiotic resistance genes spread throughout the biosphere, as well as the development of multidrug-resistant (MDR) strains, and extensively drug-resistant (XDR) strains. In the context of human and veterinary medicine, the frequent MDR exhibited by distinct strains often results in limited therapeutic alternatives for this bacterium [4,5]. Currently, only eight classes of antimicrobials are used to treat *P. aeruginosa* infections [6].

Identified more than 100 years ago, bacteriophages are being rediscovered and studied intensively now so that they can be used as an comprehensive alternative or supplement to antibiotic therapy to combat *P. aeruginosa* in humans and animals. Currently, there are over 1700 *P. aeruginosa* bacteriophage entries in the GenBank, (https://www.ncbi.nlm.nih.gov/labs/virus/vssi/#/virus?SeqType_s=Nucleotide&HostLineage_ss=Pseudomonas%20aeruginosa%20group,%20taxid:136841, search for host “Pseudomonas aeruginosa”, accessed 23 October 2025). Of the Pseudomonas aeruginosa phages with complete sequences, 60% have been identified as lytic phages, 10% as temperate (lysogenic) phages, and less than 1% as chronic phages. The remaining 29% have not yet been categorized. The most important feature of phages used in phagotherapy is a strictly lytic lifestyle to prevent the dissemination of undesirable genes from lysogenic phages. Additional requirements for therapeutic bacteriophages include an absence of toxin genes, genes that enhance pathogenicity, and genes associated with antibiotic resistance. Another requirement is the inability to perform generalized transduction. While hundreds of phages could fulfill these criteria, only a few have been tested in vivo. In the first in vivo test [7], the unclassified bacteriophage BS24 protected mice against systemic *P. aeruginosa* infections induced by injection of a lethal dose of the pathogen. The same phage protects against skin graft infections when applied to contaminated wounds in guinea-pigs [8]. The protective potential of phages was confirmed by the intraperitoneal injection of other phages (A392, or MPK1 and MPK6), all of which protected *P. aeruginosa*-infected mice [9,10]. In a veterinary clinical trial of a bacteriophage treatment for chronic infections, a mixture of six bacteriophage strains was used as a topical treatment for *P. aeruginosa* otitis in dogs, resulting in a 30.1% decrease in the clinical score and 99-fold increase in phage counts from the administered dose [11]. The pathological manifestations of lung injury in minks with hemorrhagic pneumonia caused by *P. aeruginosa* has been improved after the intranasal administration of the broad-host-range phage YH30 (genus Litunavirus) [12]. Additionally, intranasal administration of a phage protected mice with respiratory *P. aeruginosa* infection [13]. Several other phages have been documented to be active against biofilm-forming *P. aeruginosa* cells (see the review [14]). In addition to complete phages, the use of purified phage lysins to combat MDR *P. aeruginosa* strains has been reported [15,16,17].

Advantageously, the infectivity of pathogens and the activity of phages during therapy should be tested in animal models [10,18]. Lower invertebrates, such as *Caenorhabditis elegans* nematode, the fruit fly *Drosophila melanogaster*, and the wax moth *Galleria mellonella*, are easy to cultivate and cost-effective organisms that are often sensitive to pathogenic bacteria, such as *Staphylococcus aureus*, *Salmonella enteritidis*, *Clostridium difficile*, *Acinetobacter baumannii*, and *Pseudomonas aeruginosa* [19]. Previous studies have also demonstrated the feasibility of orally administering phages to adult *D. melanogaster* and the survival of phages in the gastrointestinal system [18].

The fruit fly *Drosophila melanogaster* has long been used as a model for *Pseudomonas aeruginosa* infection [20,21,22]. Dorsal thorax pricking is used for acute infection and immunological system studies, while feeding assays model chronic microbial infection [23]. *P. aeruginosa* ingested orally crosses the intestinal barrier, proliferates in the hemolymph, and accumulates in the food storage organ, the crop [24]. This causes the flies to die of bacteremia within eight days, though survival time depends on the pathogenicity of the *P. aeruginosa* isolate [25]. For example, the *chpA* mutant of *P. aeruginosa* kills flies more slowly than the PA01 strain. At the time of death, the bacterial titer reaches 1 − 40 × 10^6^ cells per fly [21]. Also, the immune system and immunological response are not well understood in *D. melanogaster*. The immune system displays similarities with the vertebrate immune system, and *D. melanogaster* exhibits evolutionary conservation of innate immune responses and NF-κB signaling cascades. Therefore, the reaction of the tested flies could predict the effectiveness on the therapeutic host in advance [22,26].

The protective effects of the MPK1 and MPK6 lytic phages were observed in a *D. melanogaster–P. aeruginosa* model with orally infected flies and 12 h of phage feeding [10]. In this study, we used bioinformatic tools to characterize six novel *Pseudomonas aeruginosa*-specific phages that were isolated from soil, wastewater, and animals. We also evaluated the therapeutic efficacy of distinct phages against *P. aeruginosa* infections in vivo using a low-cost, biological fruit fly-bacteria infection test combined with phagotherapy. These data suggest that phages can extend the flies’ lifespan and help identify the most suitable phage for potential use as a stand-alone therapy for human and animal patients with *P. aeruginosa* infections.

## 2. Materials and Methods

### 2.1. Origin of Bacteria, Virus Isolation

*P. aeruginosa* strains were obtained from the Czech Collection of Microorganisms (CCM), at Masaryk University, Brno, Czech Republic. The PAO1 type strain was obtained from the German Collection of Microorganisms and Cell Cultures (DSMZ) Leibniz Institute collection, Braunschweig, Germany (Table 1). Strain POCH2 was isolated from a canine ear swab and was identified through 16S rDNA sequencing and BLASTn comparison. All chemicals were purchased from Sigma-Aldrich, (St Louis, MO, USA). 

Soil, compost, animal feces, and wastewater samples were collected and processed as described in [27]. Briefly, one gram of the solid samples was extracted in five milliliters of Luria–Bertani broth for 16 h. The samples were then filtered through a 0.22 μm bacterial syringe filter and spotted in a 5 μL volume on the surface of soft agar containing a fresh culture of the *P. aeruginosa* POCH2 isolate. After a 16 h incubation period at 37 °C, phages from the samples producing lytic areas were passaged four times for purification. Then, they were multiplied in liquid LB broth with host bacteria and precipitated with PEG/NaCl. DNA was extracted using the phenol/chloroform method previously described in [27].

### 2.2. Phage Bioinformatics

The purified DNA was sequenced as paired-end reads on the Illumina HiSeq platform (Eurofins Genomics, Ebersberg, Germany). The raw sequence data were analyzed using CLC and Geneious Prime^®^ software, version 2025.0.3. Contigs were assembled using Tadpole, SPAdes, and Geneious algorithms. The BLAST-identified viral contigs were annotated with RAST [28]. Open reading frames were predicted using GeneMarkS v4.28 [29], and Glimmer [30] and were then manually validated using BLAST searches against the GenBank database. The online InterPro tool v. 108.0 [31] (https://www.ebi.ac.uk/interpro/search/sequence/ accessed on 11 September 2025) and HHpred version 2.08 [32] was used to predict the functions of the putative genes. A phylogenetic tree of the whole genomic sequences was constructed using ViPTree v4 with the default settings (https://genome.jp/viptree/ accessed on 11 September 2025) [33]. Sequence comparison was performed with the Global Alignment with Free End Gaps algorithm of the Geneious Prime^®^ 2025.2.2. software with a Cost Matrix of 65% similarity, a Gap Opening Penalty of 12, and a Gap Extension Penalty of 3 (default setting). The lytic/lysogenic life strategy was predicted using the phageAI server version 1.0.2 [34] and PhageLeads (https://phageleads.dk accessed on 11 September 2025) [35]. Generalized transduction was estimated based on the clustering of the large subunit of phage terminase, as described in [36].

### 2.3. Phage Biological Characteristics

The host range of the phages was determined using a spotting test. Five microliters of a serial dilution of the purified phages were spotted on soft agar containing different strains of *P. aeruginosa*, as listed in Table 1. The plates were then incubated for 16 h at 37 °C, after which the results were evaluated.

The lysis activity of the phages in liquid media was tested using an ELISA format. First, 10 µL of the purified phage (10^6^ PFU/µL) was added to 100 µL of *P. aeruginosa* POCH2 cells. Then, the mixture was incubated at 37 °C, and the optical density was evaluated at A = 600 nm. Growth curves and PFU yields were performed as previously described [37].

### 2.4. Drosophila Infectivity Test of Distinct P. aeruginosa Strains and Phage Protection

*D. melanogaster* Oregon R flies were maintained on a standard cornmeal medium at a temperature of 25 °C. Newly hatched, 3–5-day-old flies were starved for five hours at a density of 15 flies per vial. Then, the flies were transferred to a new vial containing cornmeal medium with 10^7^ CFU of *P. aeruginosa* PAO1 cells and incubated for 16 h at 25 °C. Next, the flies were then transferred to new vials containing 5% sucrose agar and approximately 10^8^ PFU of a distinct phage on filter paper. The flies were allowed to feed for 16 h at 25 °C. The infected and cured flies were then transferred to vials containing standard cornmeal medium and cultivated at 25 °C with suitable humidity. Fly mortality was monitored for 12 days, and the Kaplan–Meier survival test was used for evaluation [38]. Flies that died within five hours after manipulation were excluded from the mortality analysis. The survival data were evaluated using the log-rank test to determine statistical differences. *P*-values less than 0.05 are considered statistically significant.

The presence of bacteria in the flies was monitored in an independent assay. Individual flies were harvested, suspended in LB, and serial dilutions were inoculated onto LB plates. The presence of phages was monitored by spotting diluted homogenates on soft agar containing *P. aeruginosa* POCH2 cells [25].

## 3. Results

**Figure 1:**
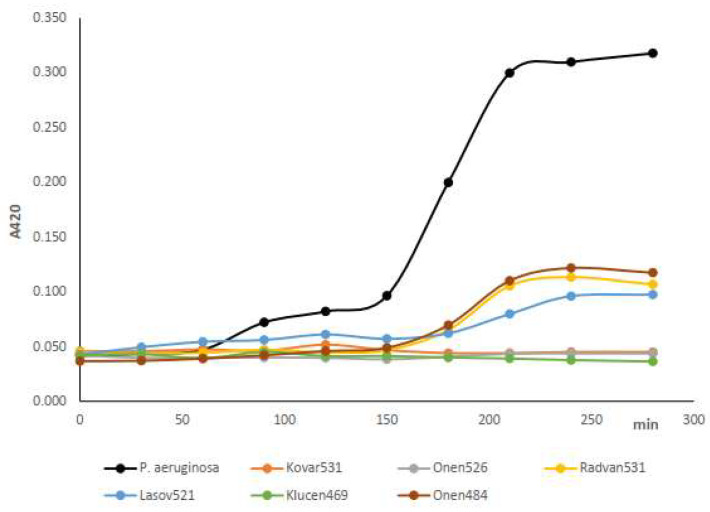
Phage effect on planktonic *P. aeruginosa* 7930 growth.

Six bacteriophages were selected from the *P. aeruginosa* POCH2 isolate due to their production of clear lytic zones. While the phages’ host ranges vary slightly, they consistently lyse six or seven out of eight tested *P. aeruginosa* strains (Table 2). The *P. aeruginosa* strain 1968 is highly resistant to the viruses and was lysed only by the Kovar531 phage. All viruses were able to suppress the planktonic growth of *P. aeruginosa* (Figure 1).

### 3.1. Molecular Characteristics of the Viruses

The natural host of these viruses is *P. aeruginosa* which has a G+C molar content of approximately 65.5%. The viruses described here have a G+C content ranging from 52.4% to 61.4%. Identities in their nucleotide sequences were revealed to be similar to those of previously described viruses; however, none are more than 95% identical to their closest relatives, indicating that they all represent new species. A whole-genome proteomic tree, constructed using the ViPTree server, classified the viruses into existing genera (Table 3). However, Klucen469 has a far lower sequence identity with its closest relatives. This suggests that it should be classified in a new genus related to the genus *Kantovirus* in the Corkvirinae subfamily.

Phage genes were predicted using Glimmer and RAST. The functions of the distinct genes were analyzed using HHpred, and InterProScan; however, a significant number of genes remain unannotated.

**Figure 2:**
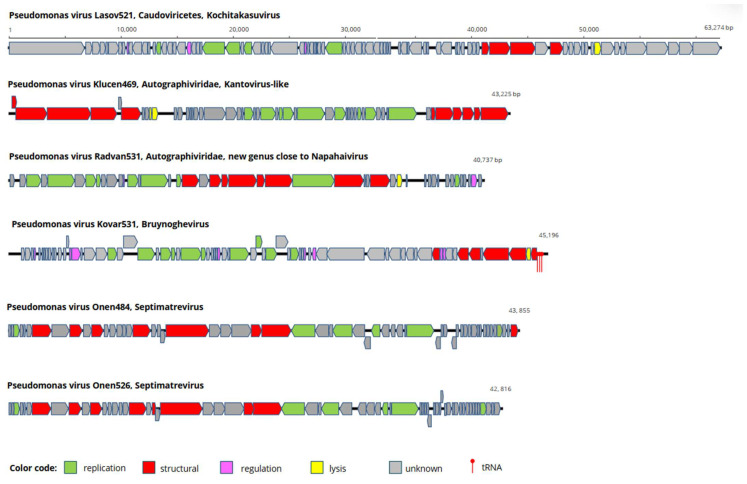
Schematic presentation of the genome arrangement of the new viruses. Structural genes are coded red, enzymes are coded green, regulatory genes are coded purple, genes involved in lysis processes are coded yellow, genes with an unknown function are coded gray, and tRNAs are marked with red dots. The genomes are drawn to scale.

The Lasov521 virus genome is 63,274 base pairs long. Eighty-seven open reading frames (ORFs) were predicted in the sense and antisense operons (Figure 2). Of a total of 87 ORFs examined, only 23 were annotated. The large and small subunits of terminase, the portal protein, and the major capsid structural protein (MCP) were identified. The DNA polymerase, helicase, and exonuclease were localized to the reverse operon. A comparison of the amino acid (aa) sequences of the MCPs of Lasov521, KPP25, R18, and YMC17 *Kochitakasuviruses* revealed that these proteins are identical in all of the viruses. Furthermore, the phage endolysin encoded by ORF79 exhibited 99.5–100% sequence identity with the aforementioned viruses. The phage integrase, which is 99% identical to the integrase of the bacterial host *P. aeruginosa*, is encoded by ORF20. This protein is necessary for integrating the phage into the host genome via site-specific recombination [39]. Similar proteins to phage integrase were also found in the genomes of other Kochitakasuviridae viruses. However, PhageLeads did not recognize genes associated with a temperate lifestyle; therefore, *Kochitakasuviruses* are expected to perform a lytic lifestyle. Large terminase amino acid sequence alignment classifies Lasov521 into the E5 cluster (see Supplementary Materials), alongside Klebsiella phage JD001, Yersinia phage PY100, and Iodobacteriophage phiPLPE [36]. These phages were predicted to have a headful packaging system and were identified as generalized transducers [5]. This feature, in conjunction with the integrase gene present in its genome, raises concerns about this phage’s suitability for curative purposes.

**Table 3:** The new *P. aeruginosa* viruses, their genome characteristics and taxonomic classification.

	Genome (bp)	ORF	G+C (%)	Mostly Related Virus	Genus	Classification	AC No:
**Klucen469**	43225	52	60.8	YMC11 NC_030923	*Kantovirus*-like	Subfamily: Corkvirinae; Order: Autographivirales	PX843241
**Radvan531**	40737	46	59.8	VSW-3 NC_041885	*Napahaivirus*-like	Family: Autonotaviridae; Order: Autographivirales	PX843244
**Kovar531**	45196	70	52.4	oldone MT119371	*Bruynoghevirus*	Class: Caudoviricetes	PX843246
**Onen484**	42855	57	53.7	Phi73 NC_007806	*Septimatrevirus*	Subfamily: Jondennisvirinae; Class: Caudoviricetes	PX843242
**Onen526**	42816	61	53.2	SCUT S4 MK165658	*Septimatrevirus*	Subfamily: Jondennisvirinae; Class: Caudoviricetes	PX843243
**Lasov521**	63274	87	60.0	KPP25 NC_024123	*Kochitakasuvirus*	Class: Caudoviricetes	PX843245

The Lasov521 virus has the highest sequence identity (93.6%) to the Pseudomonas KPP25 phage (NC_024123). According to the current taxonomic classification, Lasov521 is a new member of the *Kochitakasuvirus* genus (Caudoviricetes), alongside Pseudomonas phage R18 (NC_041964) and Pseudomonas phage YMC17/07/R4900a (OK094707). The lytic system of Lasov521 remains unclear, as only the lysozyme-like protein encoded by ORF79 and classified as a glycoside hydrolase family 19 protein has been identified in the genome.

### 3.2. Molecular Characteristics of Pseudomonas Phage Klucen469

**Figure 3:**
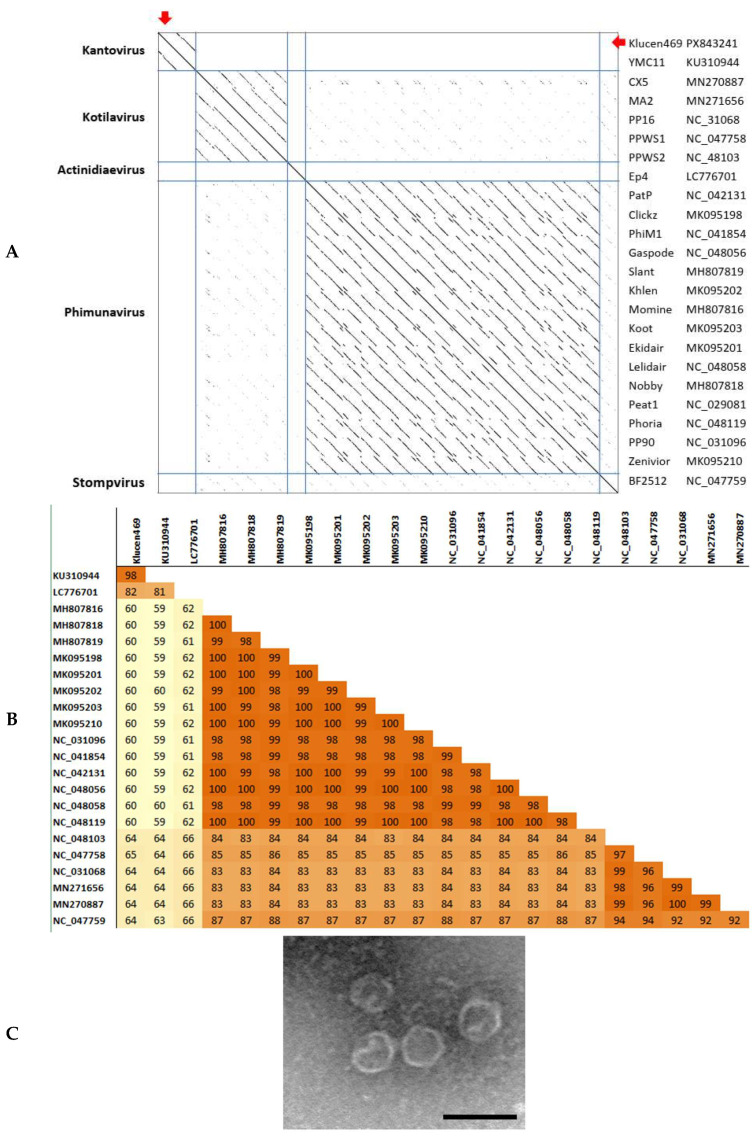
Dot plot of the concatenated genome sequences of Corkvirinae members generated by Gepard1.3 software [40]. Red arrow showed the position of Klucen469 (**A**); heat map of major capsid proteins identity of Corkvirinae members (**B**); negative-stained particles of Klucen469 observed in electron microscopy. Bar = 100 nm (**C**).

The Klucen469 phage has a 43,225 bp genome, and 52 open reading frames (ORFs) were predicted on the single strand. A total of twelve structural proteins and a lysis cassette containing a Rz-like protein, a holin, and a phage endolysin were identified. PhageLeads revealed that no genes related to the lysogeny state were present, and PhageAI also predicted a virulent lifestyle. The genome arrangement of Klucen469 resembles that of the Corkvirinae viruses. The predicted major capsid protein shares 98.5% and 82.5% identity with those of Pseudomonas phage YMC11 and Pseudomonas phage Ep4, respectively. However, the complete genome identity of the closely related Pseudomonas YMC11 *Kantovirus* is only about 81%. Therefore, it is more likely that Klucen469 will be a member of a genus that has not yet been assigned to the Corkvirinae subfamily (Figure 3A,B). The large terminase clusters with those of the bacteriophages Escherichia phage T7, Enterobacteria phage T3, and Yersinia phage YeO3-12. These bacteriophages have short direct terminal repeats on their genome ends, and natural transduction is not expected for Klucen467, therefore [5]. Electron microscopy observations revealed that Klucen469 has isometric particles with a head diameter of 68 nm ± 8 nm (n = 27) and a small tail (Figure 3C). The growth curve exhibits a lag phase of approximately 100 min, and the calculated virus yield is 52 ± 12 PFU/cell. In a double-agar plate test on sensitive hosts, the phage formed large plaques measuring 4 mm in diameter. The endolysin encoded by ORF10 is classified as an R21-like endolysin, which hydrolyzes the 1-4-β linkages between N-acetylmuramic acid and N-acetylglucosamine in the peptidoglycan molecules.

### 3.3. Molecular Characteristics of Pseudomonas Radvan531 Phage

**Figure 4:**
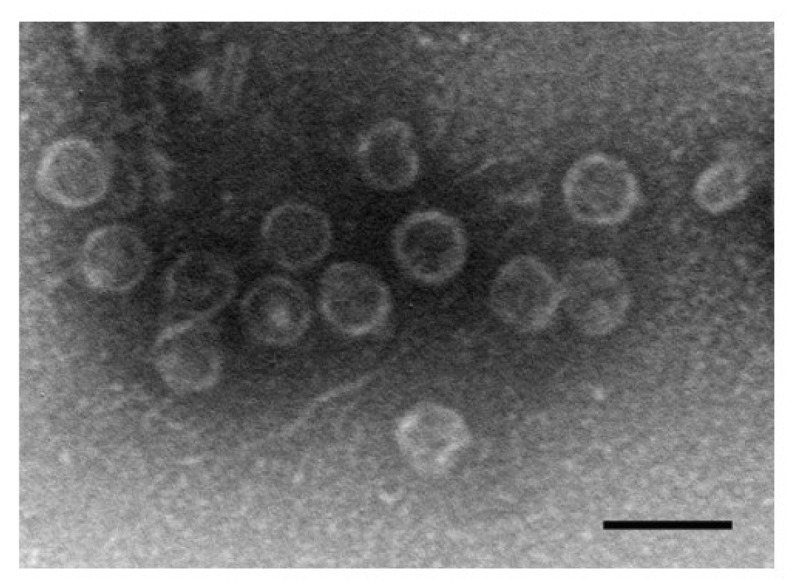
Negative stained particles of Radvan531. Bar = 100 nm.

The Radvan531 phage exhibits podovirus morphology. It has an icosahedral head measuring 52 ± 1.2 nm (n = 20) in diameter and a 6 nm tail (Figure 4). Its 40,737 bp genome has a gene arrangement similar to that of the Pseudomonas phage VSW3 napahaivirus. However, the direct nucleotide sequence identity between these two viruses is only 63% (see Supplementary Materials). Amino acid sequence identity among the structural proteins, as well as among other proteins, ranges from 36% to 88%. The major capsid protein gene exhibits 88% identity, while the large subunit of the phage terminase exhibits 77% identity. The DNA helicase has 74% identity, the DNA polymerase I has 70%; and the endolysin has 72%; with the Pseudomonas VSW3 phage endolysin. The presence of a gene encoding a single-subunit RNA polymerase indicates that Radvan531 belongs to the order *Autographivirales*. Radvan531 is more likely to be a member of an unestablished genus related to the Napahaivirus genus in the Autonotataviridae family. The Radvan531 endolysin, which is encoded by ORF33, is classified as an R21-like endolysin, similar to the one found in the Klucen469 phage.

### 3.4. Molecular Characteristics of Pseudomonas Viruses Onen484 and Onen526

The genomes of Onen484 and Onen526 are 42,855 and 42,816 nucleotides (nt) long, respectively. They have a highly similar genome arrangement and 58 and 63 predicted open reading frames (ORFs), respectively. The nucleotide sequences of the two genomes differ by 6.4%, indicating that Onen484 and Onen526 are distinct viruses. Both viruses should be classified in the *Septimatrevirus* genus and the Jondenisvirinae subfamily (See Supplementary Materials). No integrase or other proteins involved in lysogeny were identified; thus, these viruses are promising candidates for phage therapy. Furthermore, these phages likely use the headful packaging system because the large terminase subunit clusters with the E6 cluster, as well as with the Escherichia phage 933W and the Burkholderia phage Bcep22 [36]. These phages are expected to have transduction ability. The tail lysozymes (ORF3 in both phages, which are 98% identical in amino acid sequence) are likely the main lysis enzymes of these phages. These lysozymes are classified with the corresponding enzymes of Pseudomonas phages 73, Epa40, TeHO, and other Pseudomonas phages as C40 family peptidases that cleave the linkage between D-Glu and Lys within peptidoglycan stem peptides.

### 3.5. Molecular Characteristics of the Kovar531

The Pseudomonas phage Oldone (*Bruynoghevirus*, Caudoviricetes) has the highest sequence identity (91.1%) with the Pseudomonas phage Kovar531. The Kovar531 genome consists of 45,196 nucleotides (nt) and contains 68 open reading frames (ORFs) and three tRNA genes (Pro, Tyr, and Asn). In contrast, the Oldone genome contains 70 ORFs and four tRNA genes (Pro, Tyr, Asp, and Asn). Kovar531 lysozyme (ORF67) is a bacteriophage T4-like lysozyme which hydrolyzes the 1-4-β glycosidic bond between N-acetylmuramic acid and N-acetylglucosamine in peptidoglycan heteropolymers. PhageLeads found no temperate lifestyle genes and VirulenceFinder 2.0 found no virulence, exoenzyme, or antibiotic-resistance genes. Based on large terminase subunit protein sequence clustering with the E3 cluster (see the Supplementary Materials, [36]), this phage is expected to have headful packaging strategy and transduction ability.

### 3.6. Efficacy of Drosophila Flies Infection/Curation with Distinct Bacteriophage

In silico analyses, plate spot tests, and the evaluation of lysis in planktonic bacterial cultures could help predict the lytic abilities of phages. However, in vivo testing more precisely depicts this feature. While all new phages are lytic, their lytic enzymes belong to four different groups: glycoside hydrolase 19, R21-like, C40 peptidases, and T4-like. We tested the ability of the new phages to protect *D. melanogaster* flies from the fatal chronic infection caused by the wild-type strain *P. aeruginosa* PAO1 strain in vivo. Fly mortality was the only parameter tested. Four technical replicates of the infection/cure test were performed, with 15 flies each.

**Figure 5:**
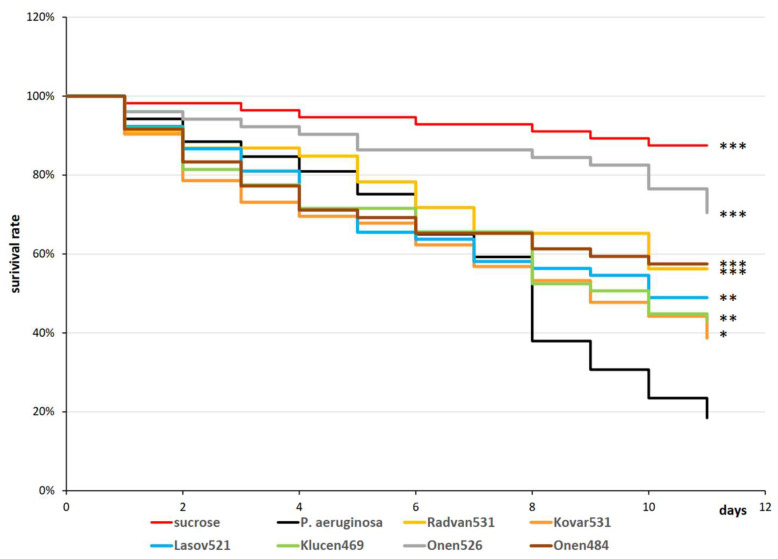
Survival assay of *D. melanogaster* (n = 60) after *P. aeruginosa* infection and following specific phage treatment. The statistical significance based on a log rank test is indicated as follows: *, *p* < 0.05, **, *p* < 0.01, ***, *p* < 0.001. Each treatment was compared to the infection control. Fifteen flies in four technical replications were analyzed.

Three- to five-day-old *D. melanogaster* flies were fed the bacterium, and then bacteriophage solutions were applied. Approximately 10^6^ CFU of *P. aeruginosa* were detected in the dead flies. However, no bacteria grew on the plates when the homogenate from the surviving flies was inoculated. The survival of the phage-curated flies was observed and calculated over time using the Kaplan–Meier test (Figure 5). While there were significant differences, all phages significantly delayed PAO1-induced killing of *D. melanogaster*. Fifty percent of the flies survived *P. aeruginosa* infection without phage curation by the 8th day. Onen526, Onen484, and Radvan531 exhibited the greatest protective capacity, achieving 50% survival after 13 days. The other phages exhibited this level of protection between days nine and 11 (Figure 5). Living phages that lyse *P. aeruginosa* in plate tests were observed in the fly homogenate after the experiment ended.

## 4. Discussion

The ever-increasing proportion of antibiotic-resistant bacteria has sparked considerable interest in alternative methods of suppressing bacterial infections and prompting numerous publications describing new bacteriophages. However, the path from describing lytic phages to applying them is long. A number of phage properties must be verified. One key feature in evaluating the therapeutic properties of phages is their ability to multiply directly within a specific organism’s bacteria. Here we describe and characterize six bacteriophages that target *P. aeruginosa* cells. All six are lytic when tested on a soft agar layer or in a liquid culture on planktonic cells. Fortunately, *P. aeruginosa* is a virulent pathogen of *Drosophila melanogaster* fruit flies. The bacteria grow exponentially within the fly, even after its death. *D. melanogaster* has been shown to be a cost-effective and manageable model for studying the treatment of broad-spectrum infections [41]. Furthermore, this model overlaps with the study of human innate immunity, as well as epithelial and muscle homeostasis [21].

Needle pricking, injector pumping, and feeding on concentrated bacteria have all been methods used. In a study of phage effectiveness, intestinal colonization more closely mimics the natural state. While the bacterium does not damage the intestine, some ingested *P. aeruginosa* cross the digestive tract and cause systemic infection [23]. In this case, the bacterium causes morbidity and mortality within two to 15 days [42,43].

Previous experiments tested six unidentified, nonclassified P. aeruginosa phages from water sources (HWPB1-3 and HWNPB1-3) for therapeutic efficacy in an acute infection model of *D. melanogaster* and in an injection-applied phage model. Therapeutic efficacy, expressed as the mean survival time of the flies, was positively correlated with the growth rate of the phages but not with their adsorption rate, lysis time, or burst size. The faster a phage grows in vitro, the more effective it is at combating bacterial infection [18]. In chronic infection with orally applied *P. aeruginosa*, both the MPK1 myovirus and the MPK6 podovirus significantly delayed the bacteria-induced killing of *D. melanogaster.* These results suggest that testing on *D. melanogaster* is valid for evaluating the efficacy of phage therapy against *P. aeruginosa* infection [10].

Recently, phages from the Septimatreviridae family have been found in hosts of the genera *Pseudomonas*, *Xanthomonas*, and *Stenotrophomonas*. The Ab26 phage only grew on the SCH strain and exhibited limited growth on several others [44]. However, the Kakheti25 phage has a very large host range [45]. The Stenotrophomonas DLP1 and DLP2 phages infect eight and nine out of 27 *S. maltophilia* strains, respectively, and can infect two separate *P. aeruginosa* strains each [46].

Phages from the *Bruynoghevirus* genus are promising candidates for phagotherapy. They form large plaques on agar plates, have a latent period of up to one hour, and produce more than 100 PFU per cell. Additionally, they exhibit lytic activity against planktonic and biofilm cells. Their host range is 45–72%, and their genomes lack sequence similarity to genes that encode integrases, recombinases, and repressors of the lytic cycle. Therefore, they are considered obligatory lytic [47,48,49]. Pa222 and Pa223, which target *P. aeruginosa*, have previously been used for human phage therapy [50].

Phagotherapy should be performed using the best available phages. To improve the lytic efficacy of the phages, they can be adapted to the targeted strain via at least 15 serial passages could be performed [51]. However, this process cannot always be applied. For example, in animal phagotherapy with broiler chickens, there is insufficient time to precisely analyze the pathogenic bacterial strain present in the animals and perform the phage adaptation process when phages are administered prophylactically during the first days of life. This process takes a minimum of five days [52], not to mention preparing the necessary amount of application phage.

*Pseudomonas aeruginosa* is an important pathogen for humans and animals, and significant efforts have recently been made to identify specific bacteriophages. We supplement bioinformatic data on phage genomes with a relatively quick and low-cost biological test using *P. aeruginosa*-sensitive *Drosophila* flies to study the protective effects of different phages against *P. aeruginosa*. While the quantity of bacteria and phages ingested by each fly is not under experimental control, the experiment provides an unambiguous count of dead and surviving flies that can be statistically analyzed. This enables us to quickly select the most promising phage/bacteria, faster than by experimentally analyzing the lytic enzymes of the phages.

## Figures and Tables

**Table 1:** Bacteria used in this study. CCM-Czech Collection of Microorganisms, Masaryk University, Brno, Czech Republic; DSMZ-German Collection of Microorganisms and Cell Cultures, GmBH, Leibniz Institute, Braunschweig, Germany.

Bacteria/Strain	Collection	Source
*P. aeruginosa* POCH2	-	canine ear swab
*P. aeruginosa* 1959	CCM	urine
*P. aeruginosa* 1961	CCM	outer-ear infection
*P. aeruginosa* 1968	CCM	unknown source
*P. aeruginosa* 3630	CCM	otitis externa
*P. aeruginosa* 3989	CCM	waterworth, clinical
*P. aeruginosa* 7930	CCM	animal room water
*P. aeruginosa* 22644	DSMZ	infected wound, type strain

**Table 2:** Host range and reactivity of distinct viruses. + clear zone on distinct host, - no lysis.

Bacteria Strain	Klucen469	Kovar531	Radvan531	Onen484	Onen526	Lasov521
*P. aeruginosa* POCH2	+	+	+	+	+	+
*P. aeruginosa* 1959	+	+	+	+	+	+
*P. aeruginosa* 1961	+	+	-	-	-	+
*P. aeruginosa* 1968	-	+	-	-	-	-
*P. aeruginosa* 3630	+	-	+	+	+	+
*P. aeruginosa* 3989	+	+	+	+	+	+
*P. aeruginosa* 7930	+	+	+	+	+	+
*P. aeruginosa* 22644	+	+	+	+	+	+

## Data Availability

The complete genome sequence of the Pseudomonas viruses Klucen469, Radvan531, Kovar531, Onen484, Onen526, and Lasov521 have been deposited in GenBank under accession numbers PX843241–PX843246.

## Supplementary Materials

The following supporting information can be downloaded at: https://www.mdpi.com/article/10.3390/pathogens15040411/s1.
