# Supplementary material for: Antibacterial Efficacy of Pseudomonas aeruginosa Bacteriophages on a Drosophila Infection Model
Source: Pathogens. 2026 Apr 10;15(4):411. doi: 10.3390/pathogens15040411 (PMC13119210; doi:10.3390/pathogens15040411)

Article

Antibacterial efficacy of *Pseudomonas aeruginosa* bacteriophages on a *Drosophila* infection model

Karel Petrzik [1,*], Sára Brázdová [1]

Supplementary material

1. ViPTree proteomic trees with distinct new viruses
2. Reconsctruction of N-J trees of large terminase proteins
3. Global genomic alignment with the closest viruses

1. ViPTree of the viruses, global genomic sequence identity to related viruses

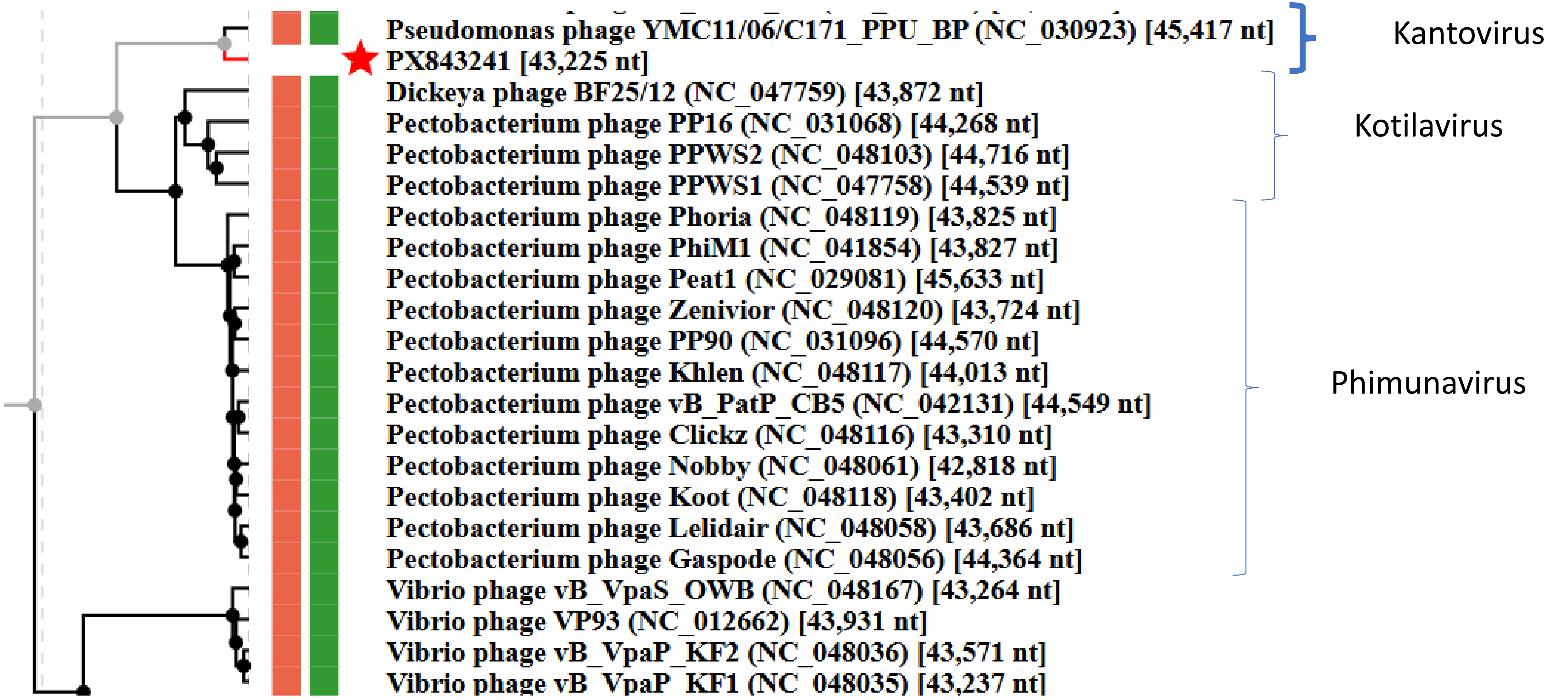

PX843241 identity to YMC11 = 80.8%

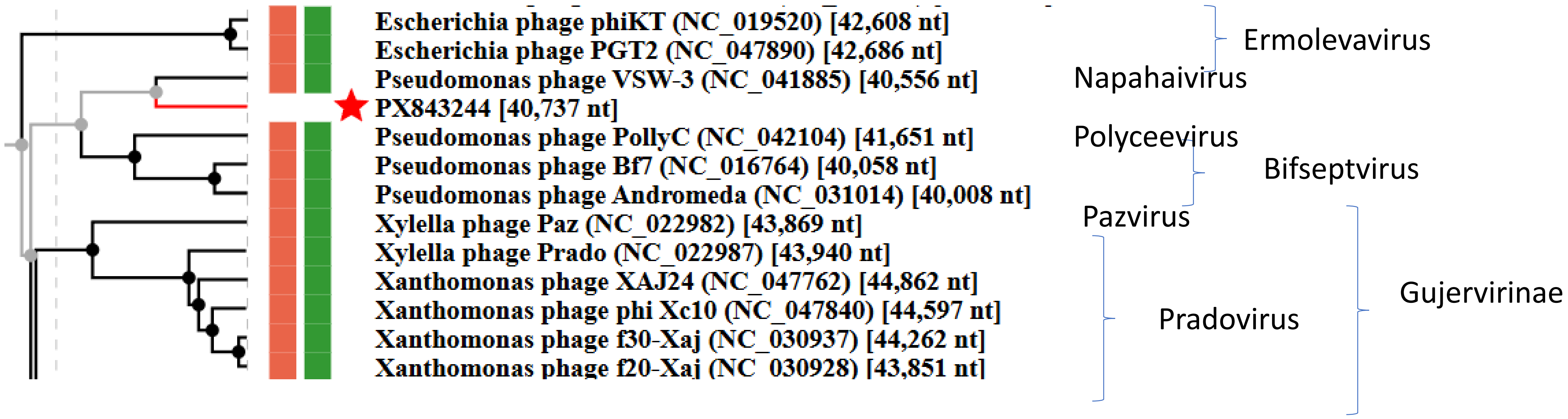

PX843244 identity to VSW-3 = 63.0%
to PollyC = 55.3%
to Bf7 = 55.4%

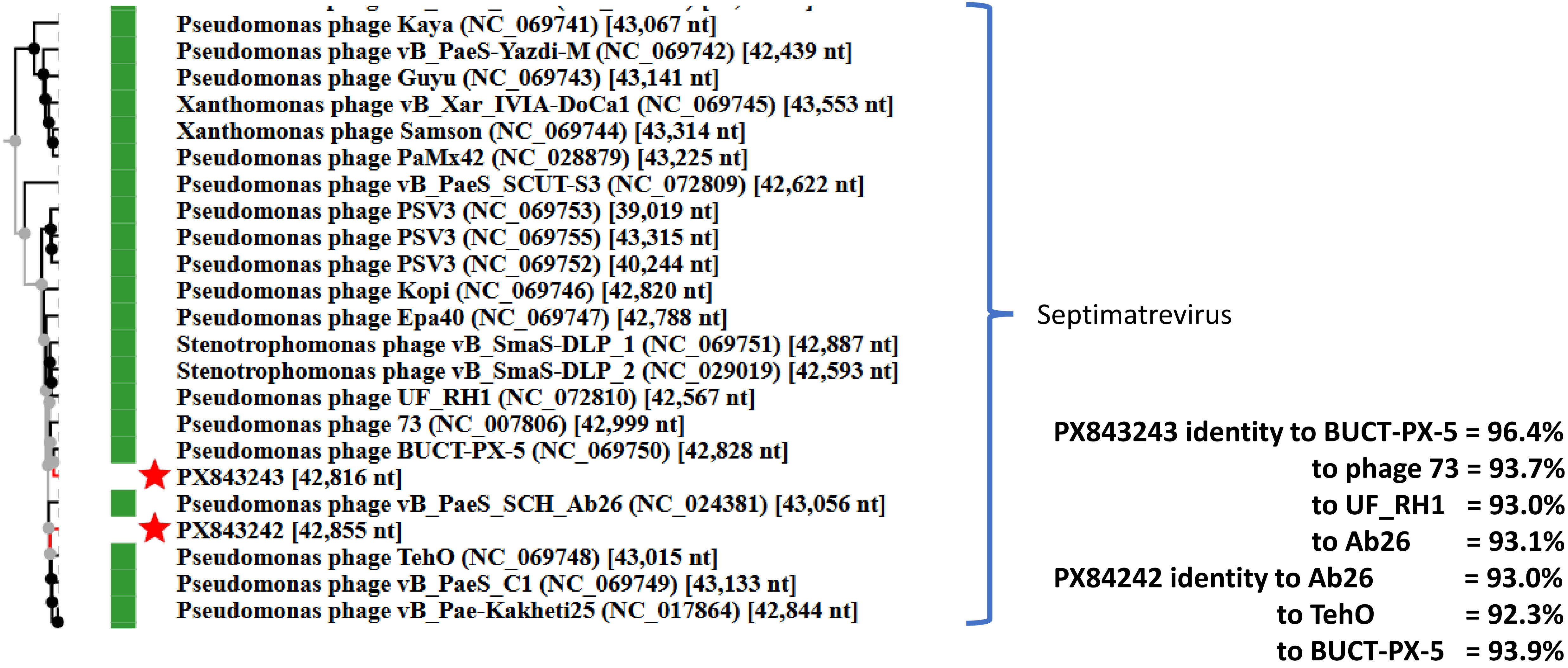
Pseudomonas phage Kaya (NC_069741) [43,067 nt]
Pseudomonas phage vB_PaeS-Yazdi-M (NC_069742) [42,439 nt]
Pseudomonas phage Guyu (NC_069743) [43,141 nt]
Xanthomonas phage vB_Xar_IVIA-DoCa1 (NC_069745) [43,553 nt]
Xanthomonas phage Samson (NC_069744) [43,314 nt]
Pseudomonas phage PaMx42 (NC_028879) [43,225 nt]
Pseudomonas phage vB_PaeS_SCUT-S3 (NC_072809) [42,622 nt]
Pseudomonas phage PSV3 (NC_069753) [39,019 nt]
Pseudomonas phage PSV3 (NC_069755) [43,315 nt]
Pseudomonas phage PSV3 (NC_069752) [40,244 nt]
Pseudomonas phage Kopi (NC_069746) [42,820 nt]
Pseudomonas phage Epa40 (NC_069747) [42,788 nt]
Stenotrophomonas phage vB_SmaS-DLP_1 (NC_069751) [42,887 nt]
Stenotrophomonas phage vB_SmaS-DLP_2 (NC_029019) [42,593 nt]
Pseudomonas phage UF_RH1 (NC_072810) [42,567 nt]
Pseudomonas phage 73 (NC_007806) [42,999 nt]
Pseudomonas phage BUCT-PX-5 (NC_069750) [42,828 nt]
PX843243 [42,816 nt]
Pseudomonas phage vB_PaeS_SCH_Ab26 (NC_024381) [43,056 nt]
PX843242 [42,855 nt]
Pseudomonas phage TehO (NC_069748) [43,015 nt]
Pseudomonas phage vB_PaeS_C1 (NC_069749) [43,133 nt]
Pseudomonas phage vB_Pae-Kakheti25 (NC_017864) [42,844 nt]
Septimatrevirus
PX843243 identity to BUCT-PX-5 = 96.4%
to phage 73 = 93.7%
to UF_RH1 = 93.0%
to Ab26 = 93.1%
PX84242 identity to Ab26 = 93.0%
to TehO = 92.3%
to BUCT-PX-5 = 93.9%

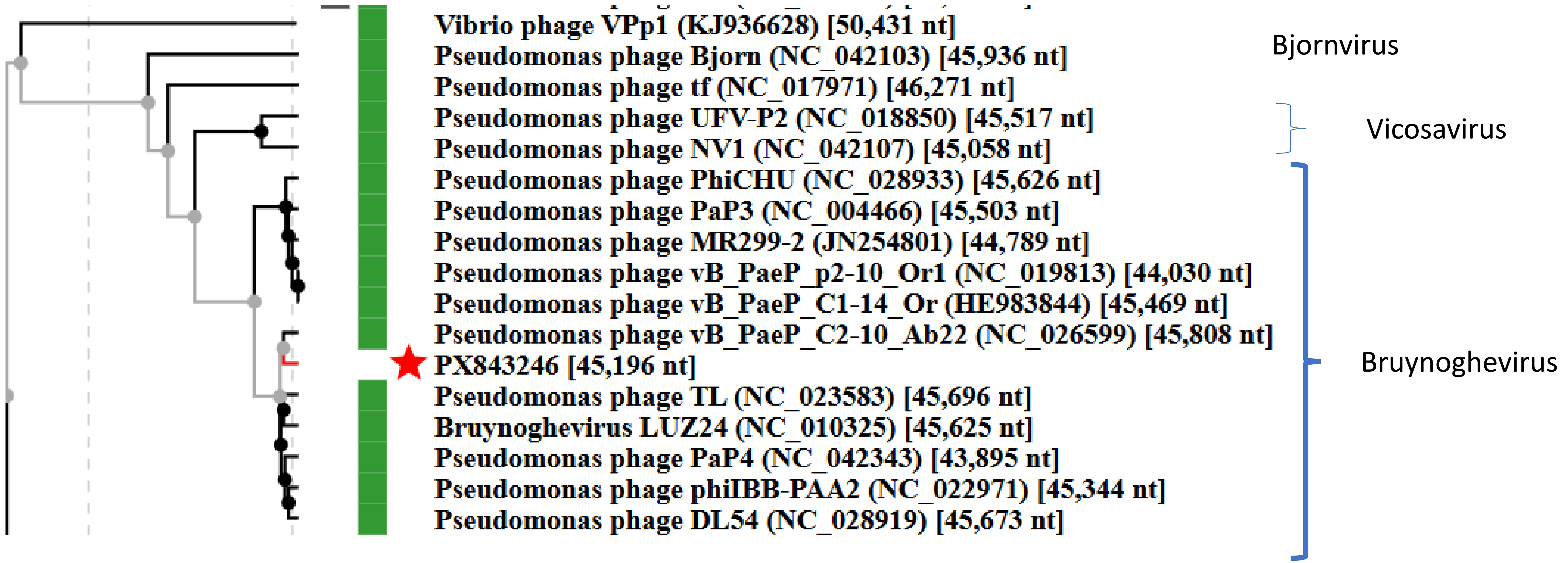

PX843246 identity to oldone = 91.1.%
to Ab22 = 90.9%
to TL = 88.3%
to LUZ24 = 84.0%

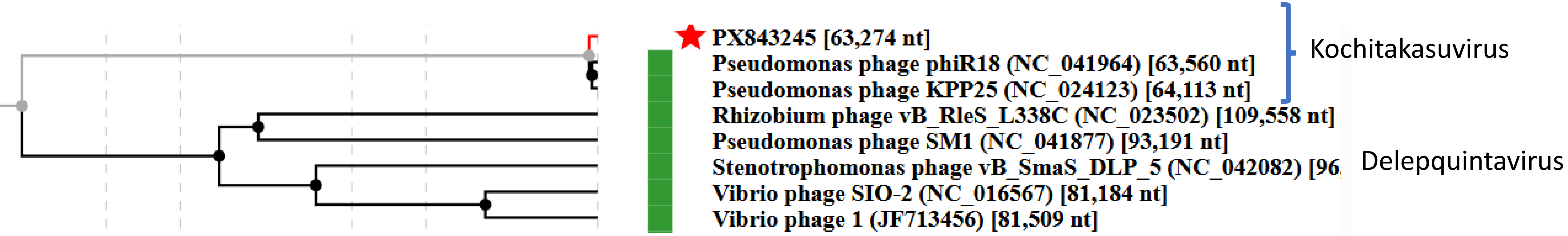

PX843245 identity to R18 = 92.7%
to KPP25 = 93.6%

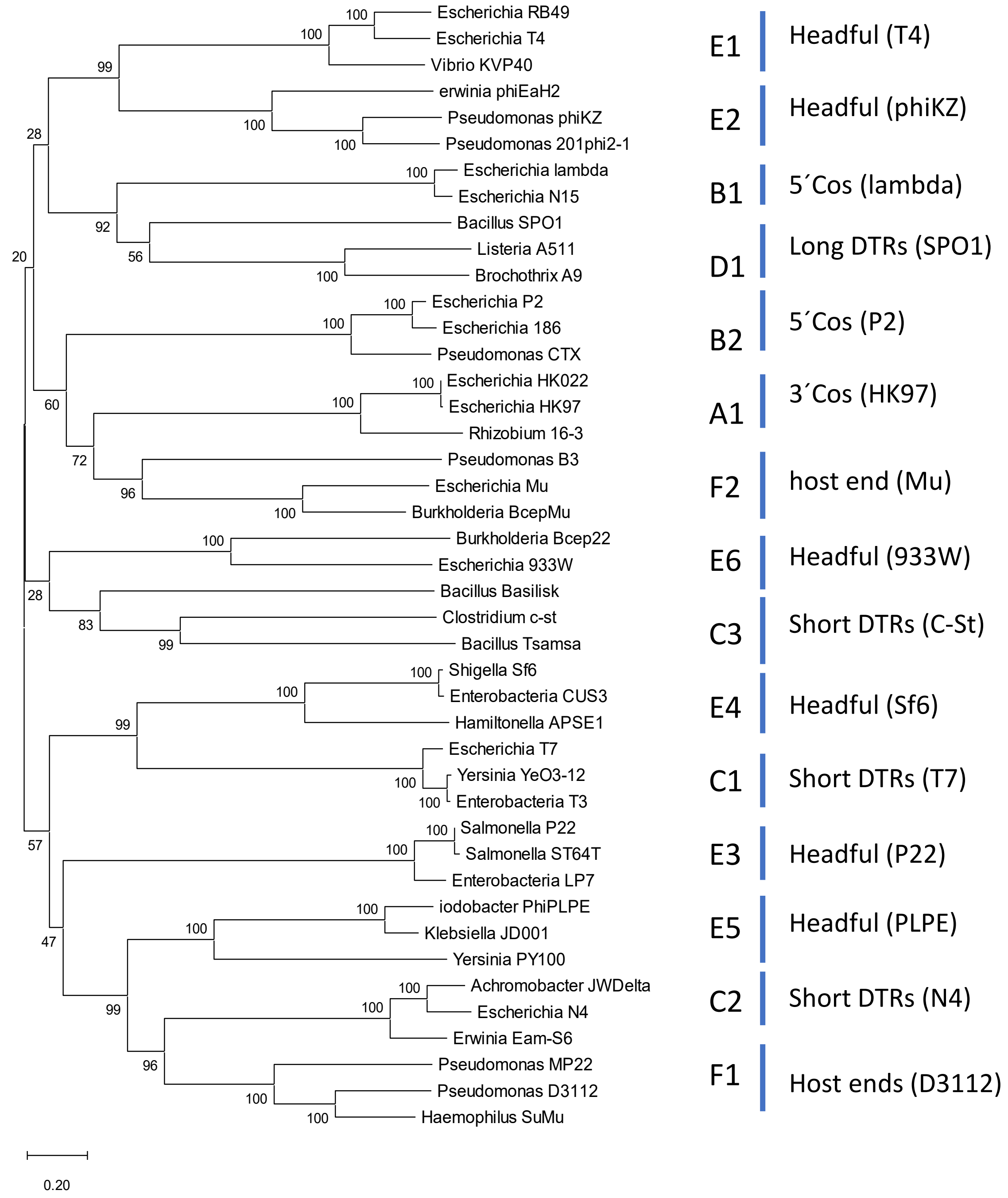

2. Reconstruction of N-J tree of large terminase proteins

The tree was generated in MEGA12, ClustalW alignment with BLOSOM protein weight matrix; N-J tree with poison correction model; bootstrap values are for 1000 trials.

Sequences as published in Merill et al. 2016 are used.

Packaging mechanisms, cluster numbers, and physical ends are marked.

Klucen469 large terminase clustering

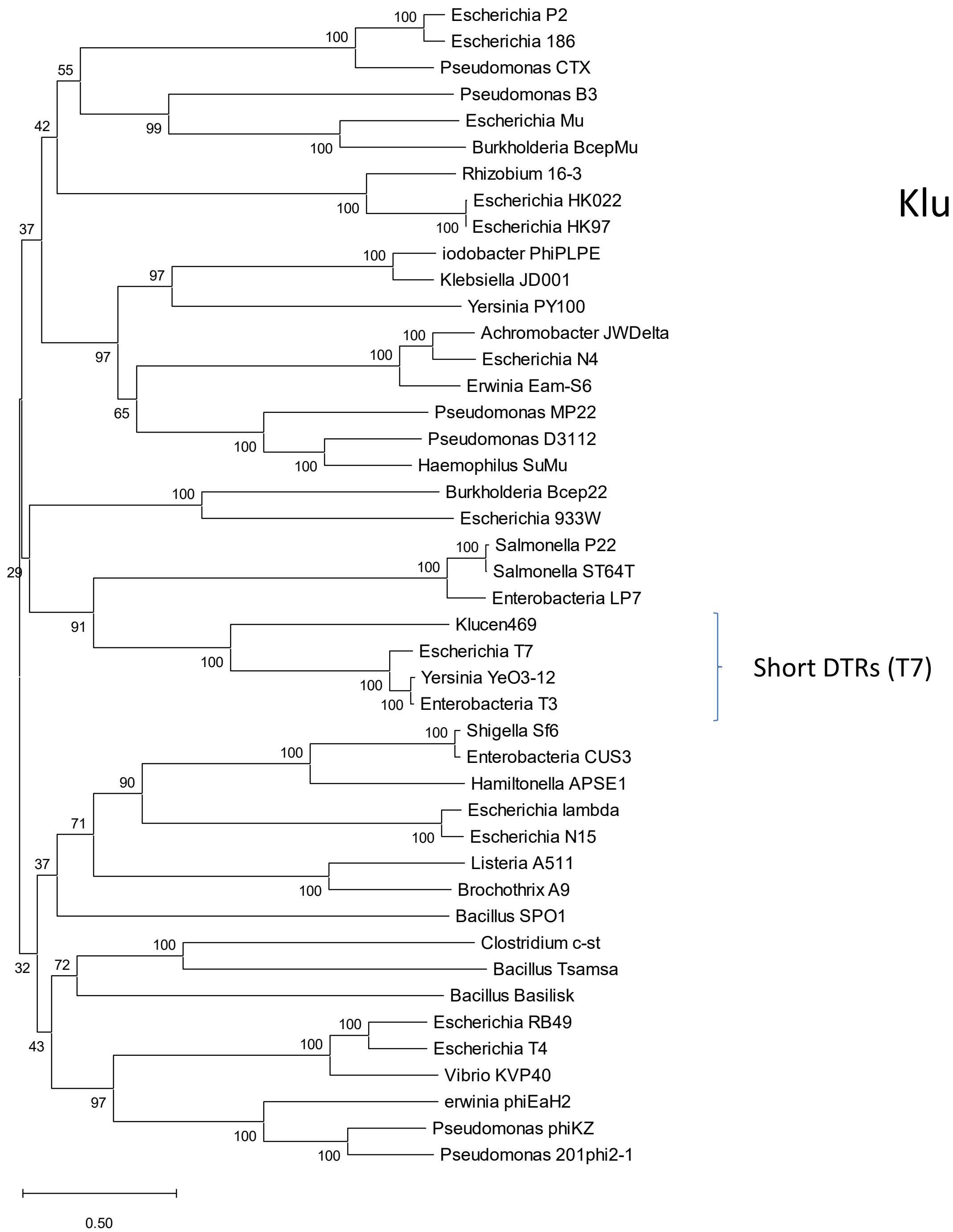

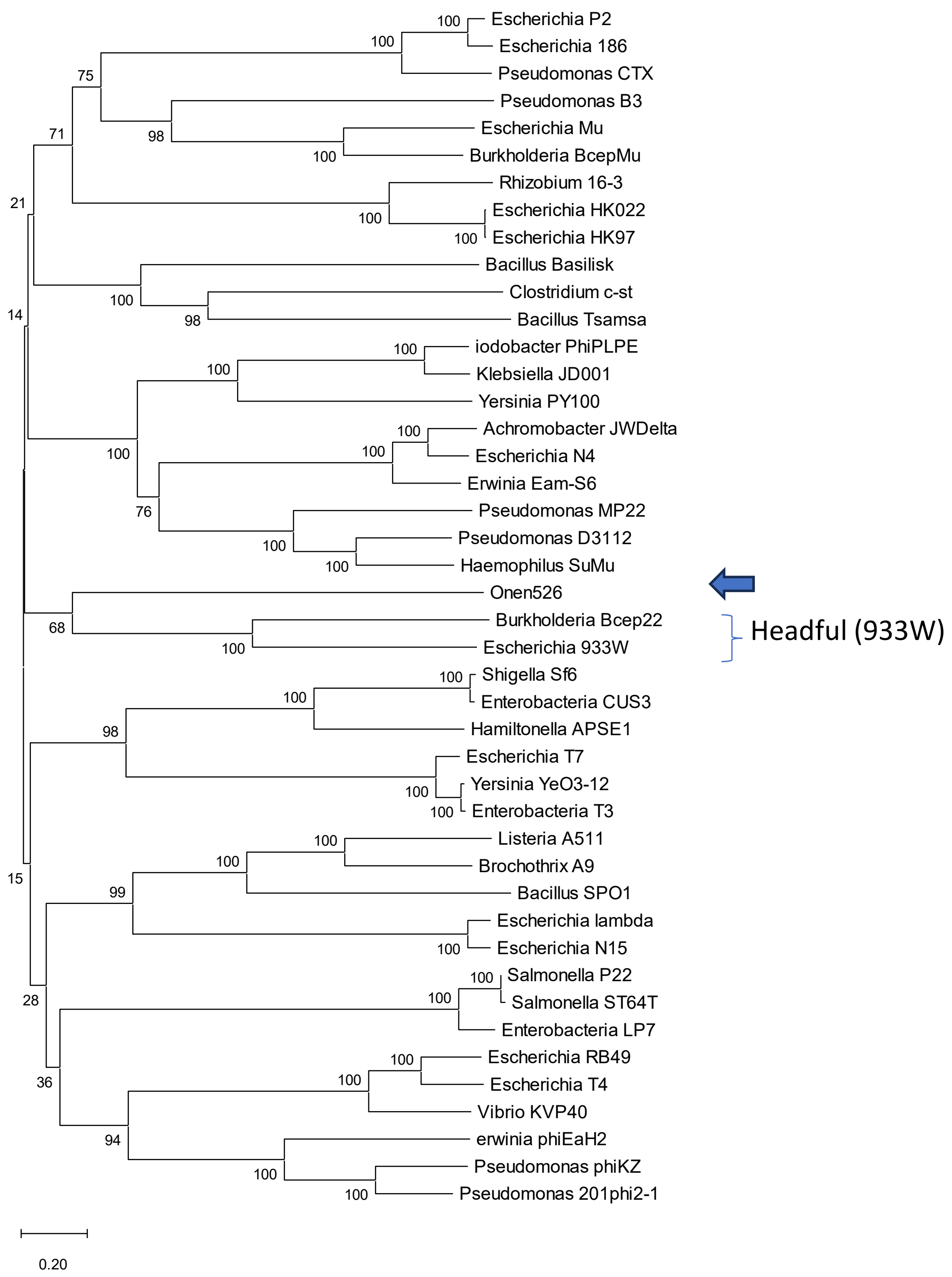

Onen526 and Onen484 large terminase clustering – probably headful

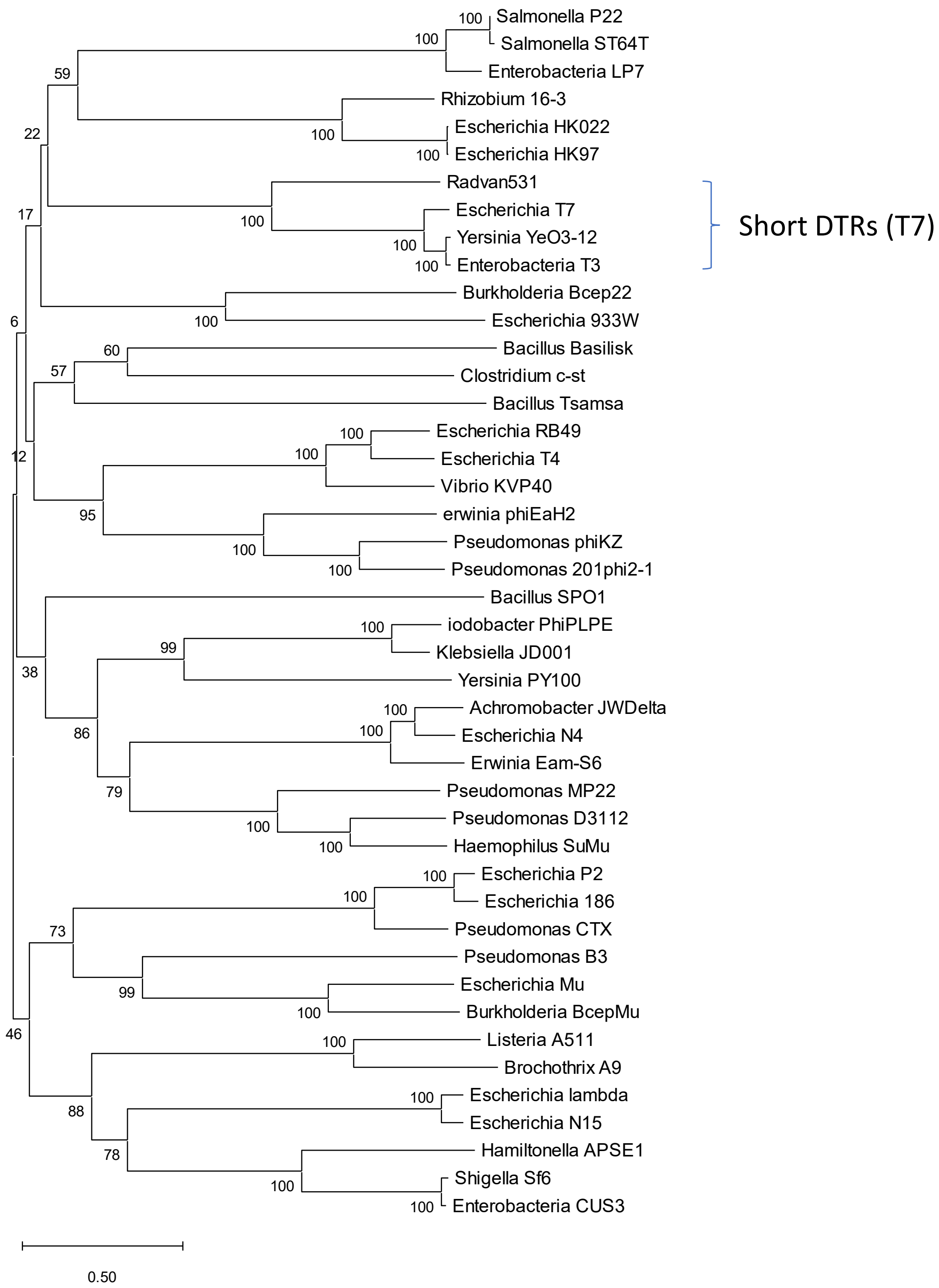

Radvan531 large terminase clustering – probably short DTRs

Lasov521 large terminase clustering

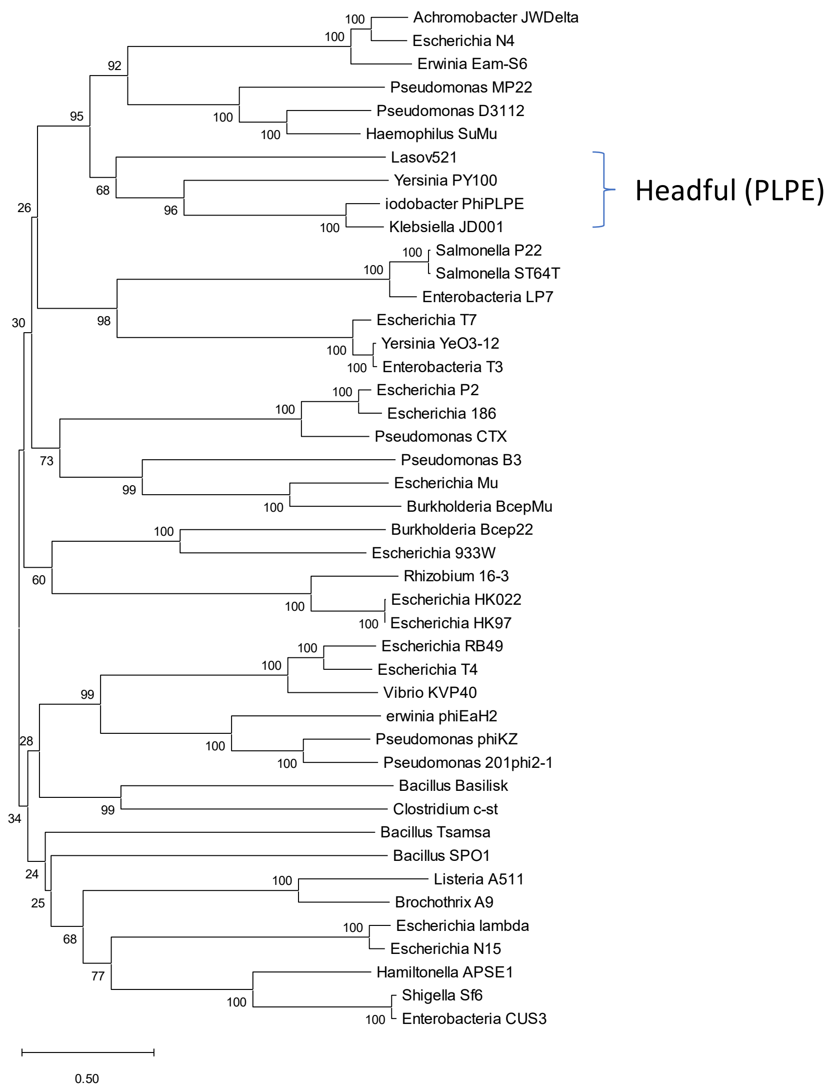

Kovar531 large terminase clustering

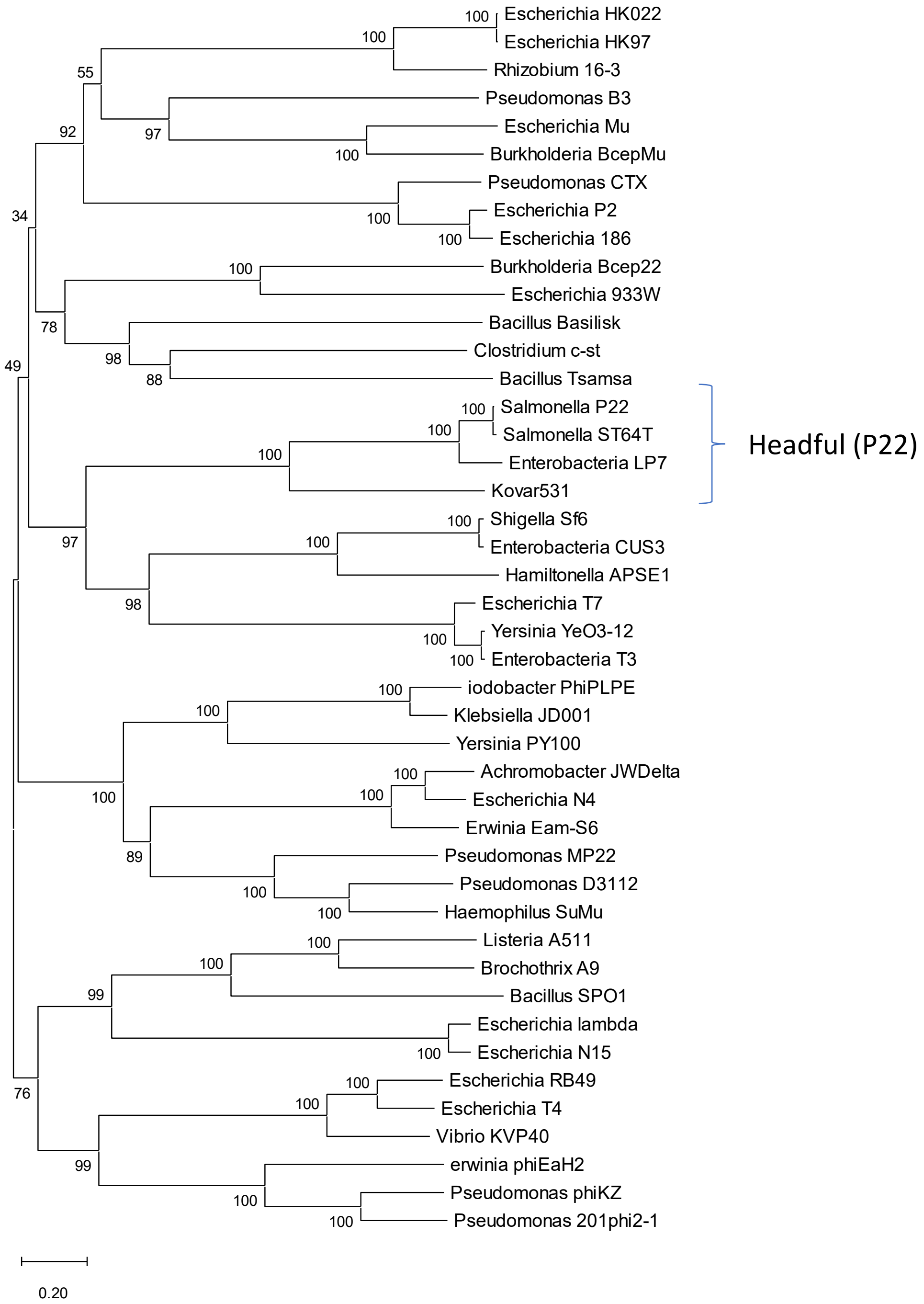

3. Global genomic alignment with the closest viruses

The sequences orientation and the starting point were corrected and sequences were aligned with the Global Alignment with Free End Gaps algorithm from the Geneious Prime v. 2025.2.2. software with a Cost Matrix of 65 % similarity, a Gap Opening Penalty of12, and a Gap Extension Penalty of 3 (default setting).

PX843244 (Radvan531) vz NC_041885 (VSW3) **63% sekvence identity**

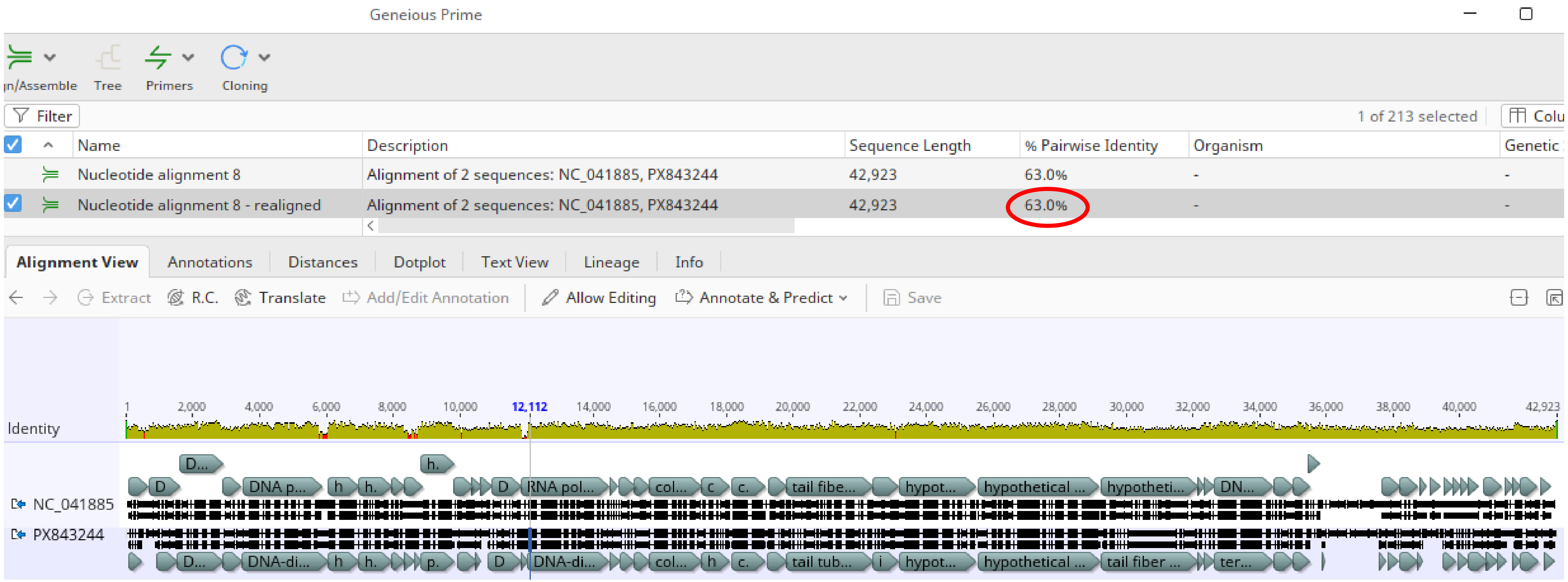

PX843244 (Radvan531) vz NC_042104 (pollyC) – **sequence identity 55.3%**

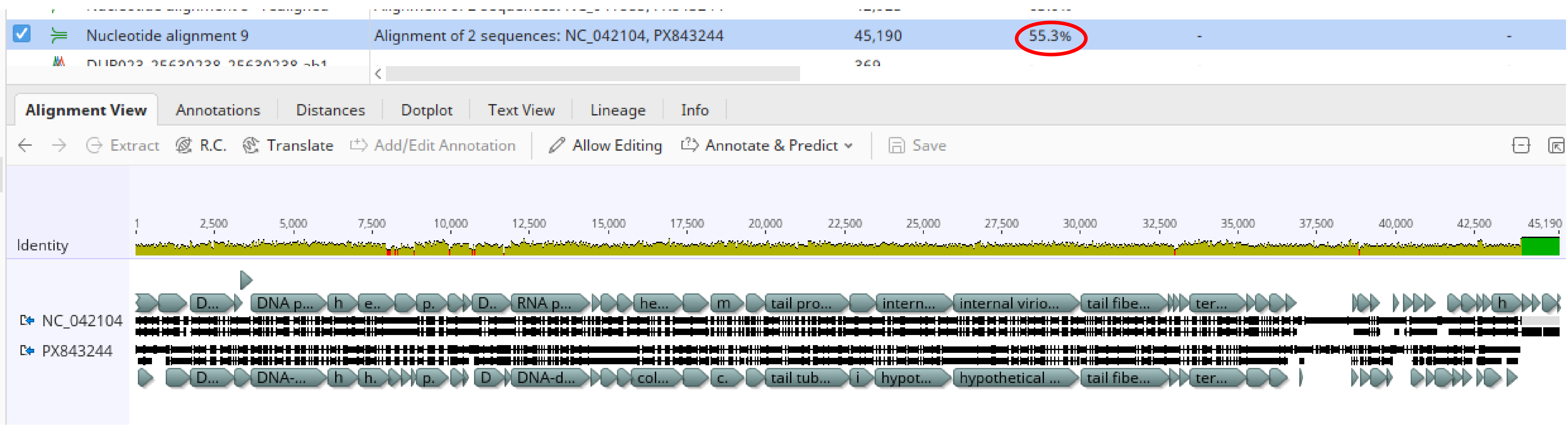

PX843241 (Klucen469) with NC_030923 (YMC11) – **sequence identity 80.8%**

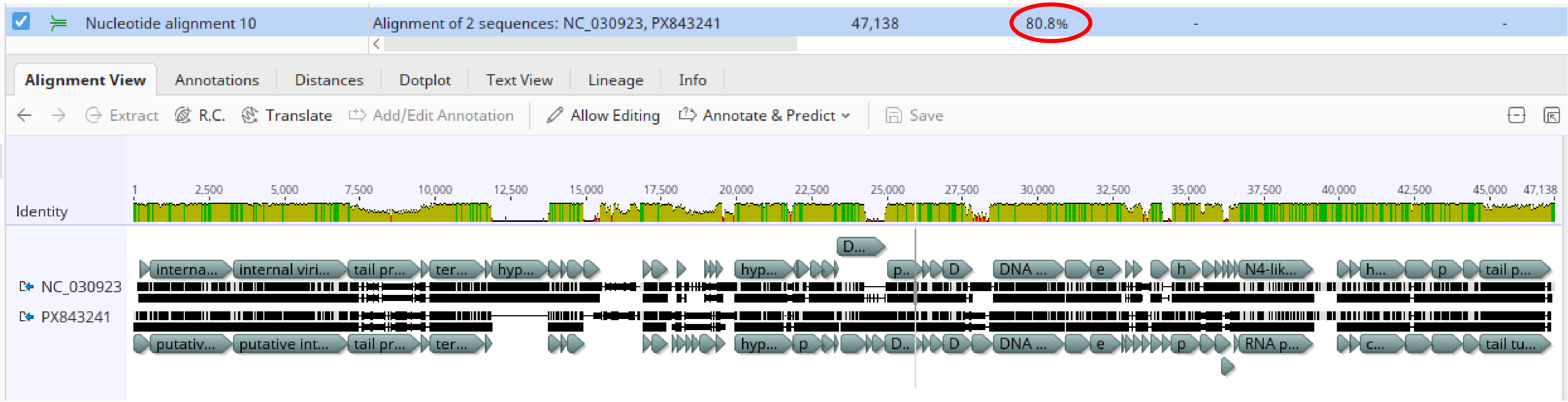

PX843242 (Onen484) with NC_069750 (BUCT-Px-5) – **sequence identity 93.9%**

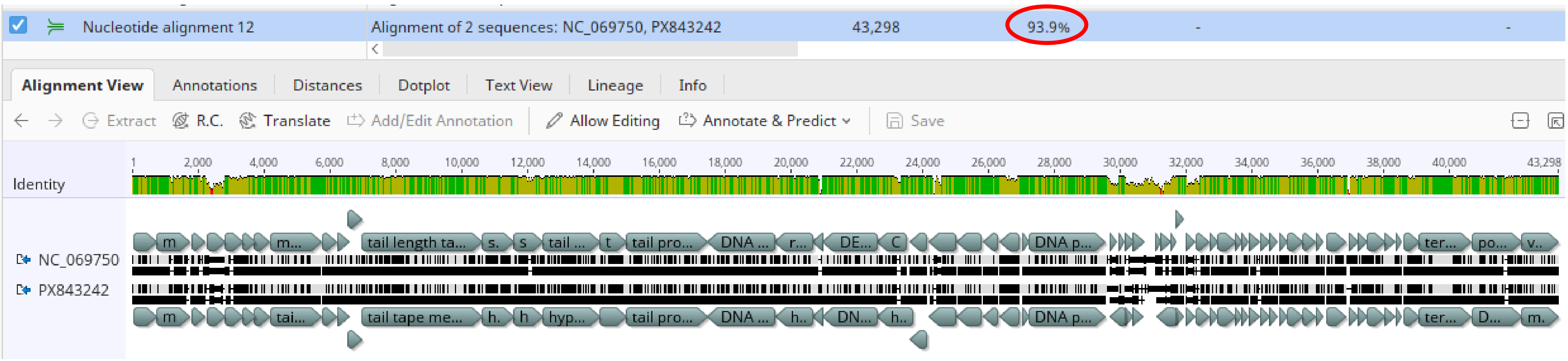

PX843243 (Onen526) with NC_069750 (BUCT-Px-5) – **sequence identity 96.4% (strain of BUCT-Px-5)**

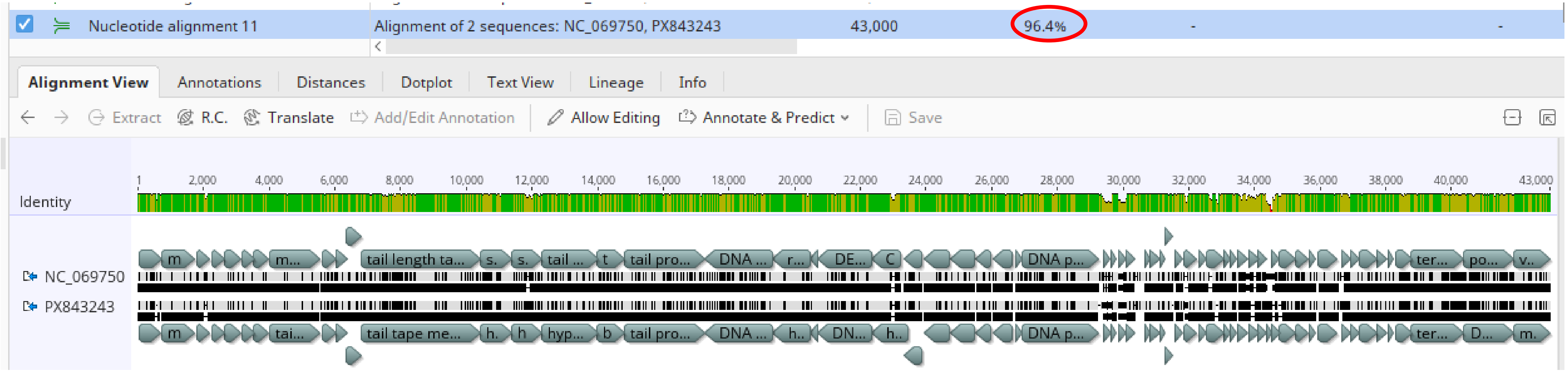

PX843245 (Lasov521) with NC_024123 (KPP25) – **sequence identity 93.5%**

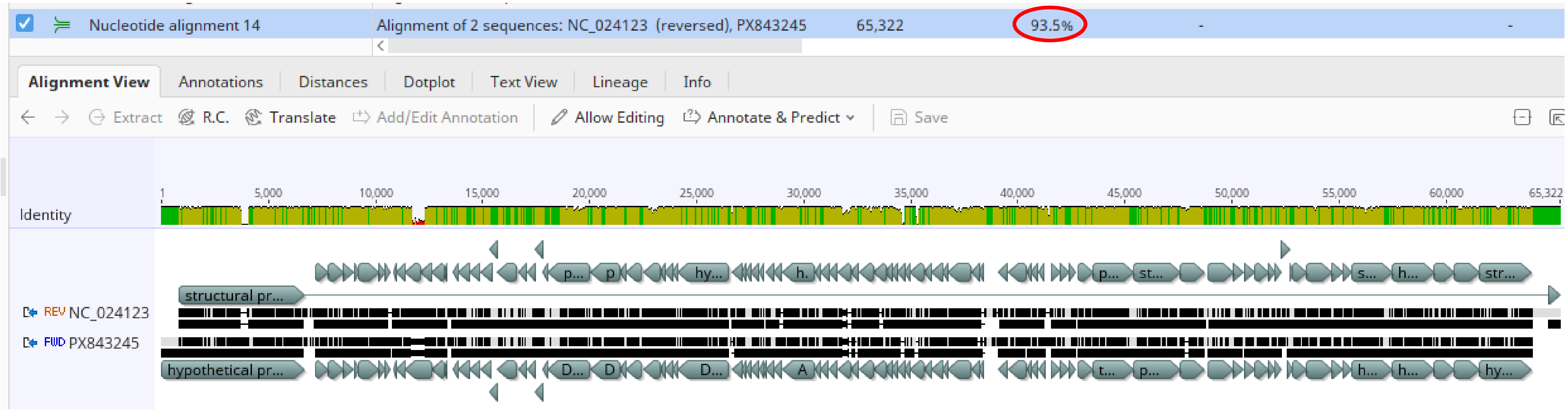

PX843246 (Kovar531) with MT119371 (oldone) – **sequence identity 91.1%**

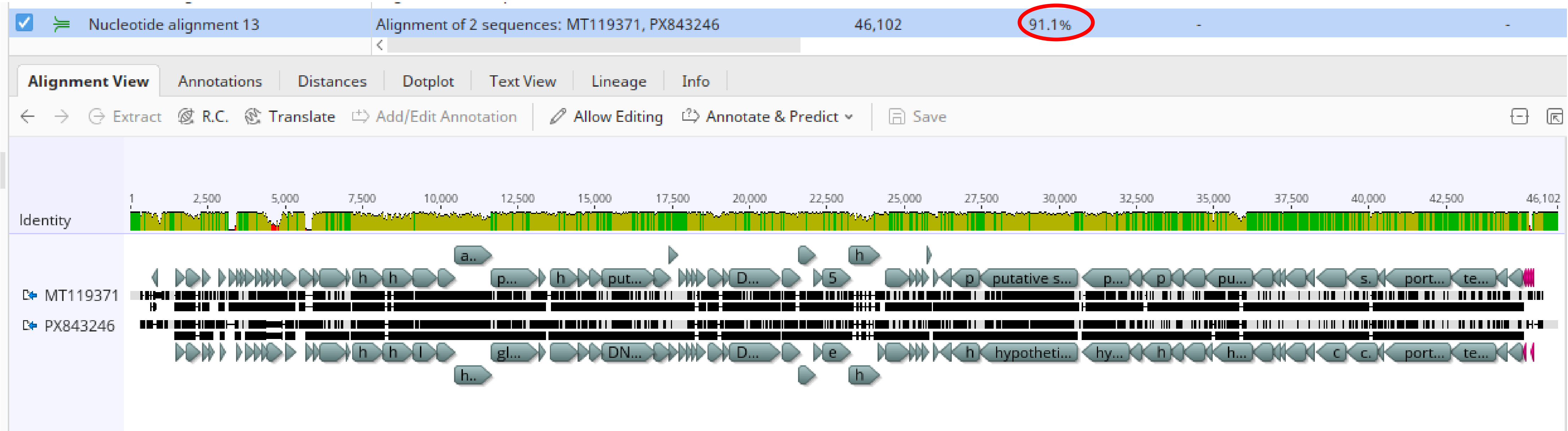

Supplement: Supplementary file 1 [file pathogens-15-00411-s001.zip › pathogens-4199483-supplementary.pdf]
